# Prognostic Power of a Chaperonin Containing TCP-1 Subunit Genes Panel for Hepatocellular Carcinoma

**DOI:** 10.3389/fgene.2021.668871

**Published:** 2021-04-08

**Authors:** Wenli Li, Jun Liu, Hetong Zhao

**Affiliations:** ^1^Reproductive Medicine Center, Yue Bei People's Hospital, Shantou University Medical College, Shaoguan, China; ^2^Department of Traditional Chinese Medicine, Changhai Hospital, Naval Military Medical University, Shanghai, China

**Keywords:** chaperonin containing T-complex protein 1, hepatocellular carcinoma, prognostic signature, prognostic factor, immune infiltration

## Abstract

Chaperonin containing TCP-1 (T-complex protein 1) (CCT) is a large molecular weight complex that contains nine subunits (TCP1, CCT2, CCT3, CCT4, CCT5, CCT6A, CCT6B, CCT7, CCT8). This study aimed to reveal key genes which encode CCT subunits for prognosis and establish prognostic gene signatures based on CCT subunit genes. The data was downloaded from The Cancer Genome Atlas, International Cancer Genome Consortium and Gene Expression Omnibus. CCT subunit gene expression levels between tumor and normal tissues were compared. Corresponding Kaplan-Meier analysis displayed a distinct separation in the overall survival of CCT subunit genes. Correlation analysis, protein-protein interaction network, Gene Ontology analysis, immune cells infiltration analysis, and transcription factor network were performed. A nomogram was constructed for the prediction of prognosis. Based on multivariate Cox regression analysis and shrinkage and selection method for linear regression model, a three-gene signature comprising CCT4, CCT6A, and CCT6B was constructed in the training set and significantly associated with prognosis as an independent prognostic factor. The prognostic value of the signature was then validated in the validation and testing set. Nomogram including the signature showed some clinical benefit for overall survival prediction. In all, we built a novel three-gene signature and nomogram from CCT subunit genes to predict the prognosis of hepatocellular carcinoma, which may support the medical decision for HCC therapy.

## Introduction

Hepatocellular carcinoma (HCC) is the seventh-most common tumor and the third leading cause of cancer-related death around the world (Bray et al., 2018). Surgical treatment is the most commonly used curative approach, but its benefits are negligible (Yan et al., 2016). HCC is also a kind of biological heterogeneous malignancy whose cellular and molecular mechanisms are rarely realized, limiting the improvement of the therapy efficacy (Erstad et al., 2018). Thus, the identification of molecular biomarkers is urgently needed to improve prognosis prediction, and assist the development of possible novel therapies.

High-throughput genomic studies of HCC have provided a comprehensive view of HCC thus far (Cancer Genome Atlas Research Network., 2017). Numerous studies have identified several biological pathways that may be targeted in the development of HCC, and genetic analysis have identified somatic mutations in multiple genes correlated to HCC (Pogribny and Rusyn, 2014; Ganly et al., 2018). It was found that the survival of patients with HCC was related to several genes through bioinformatic analysis (Cheng et al., 2018; Bayo et al., 2019; Yan et al., 2019). However, these studies had some limitations, including small populations, and lack of validation.

Chaperonin containing TCP-1 [TCP (T-complex protein 1) ring complex (TRiC), CCT/TRiC] is a large protein complex in eukaryotic cells that is composed of eight homologous subunits (TCP1, CCT2, CCT3, CCT4, CCT5, CCT6, CCT7, CCT8). The eight subunits are formed by two stacked aromatic rings as CCT 1-4-2-5-7-8-6-3-(1), which form a cage (Ditzel et al., 1998; Araki et al., 2016). Furthermore, CCT6 comprises two subunits: 6A and 6B. The CCT complex could assist the regulates telomere maintenance, folding of proteins, actin, and tubulin (Freund et al., 2014). Independent genes on separate chromosomes encode the nine subunits. The functions of CCT subunits have been studied in various tumors such as breast cancer (Guest et al., 2015; Bassiouni et al., 2016), esophageal squamous (Yang et al., 2018), thyroid neoplasia (Belousov et al., 2015), and glioma (Qiu et al., 2015). Moreover, recent studies have shown that CCT3, CCT7, and CCT8 were associated with HCC progression (Huang et al., 2014; Cui et al., 2015; Gao et al., 2019). As we have seen, little is known about the prognostic value of CCT subunit genes in HCC. Therefore, our study aims to identify the relationship between CCT subunit genes expression levels and HCC prognosis.

## Materials and Methods

### Patients and Samples Acquisition

Clinical and genomic data of HCC cohorts were downloaded from The Cancer Genome Atlas (TCGA-LIHC, http://cancergenome.nih.gov/), International Cancer Genome Consortium (ICGC-LIRI-JP, https://www.icgc.gov/), and Gene Expression Omnibus (GEO-GSE14520, https://www.ncbi.nlm.nih.gov/geo/) data portal in June 2019. All samples from the TCGA dataset were randomly assigned to a training set (50%) and a validation set (50%). The samples from the ICGC and GSE14520 cohort were regarded as the testing set.

### Functional Analysis and Protein-Protein Interaction Network Construction

Gene Ontology (GO) analysis of CCT subunit genes was performed to identify the molecular function, biological processes, and cellular components using clusterProfiler R package. The top ten gene ontology terms were chosen with false discovery rate (FDR) <0.05. The protein-protein interaction (PPI) network and significant signaling pathways of CCT subunit genes were investigated, using GeneMANIA database (http://genemania.org) (Warde-Farley et al., 2010).

### Immune Cells Infiltration Analysis

TIMER database (https://cistrome.shinyapps.io/timer/) (Li et al., 2017) was used to assess the associated between the expression of CCT subunit genes and six immune cells infiltration, including B cell, CD4+ T cell, CD8+ T cell, Macrophage, Neutrophil, and Dendritic cell.

### Transcription Factor Network

ChEA3 is a transcription factor (TF) prediction database (https://maayanlab.cloud/chea3/), which integrates ENCODE, ReMap and some independent published CHIP-seq data (Keenan et al., 2019). In this study, we predict the CCT subunit genes-regulating TF based on the ChEA3 database. Next, the ranked top 30 TFs were selected for survival analysis by univariate Cox regression. Furthermore, we constructed a transcription factor regulatory network.

### CCT Subunit Genes Signature Identification and Validation

Based on the CCT subunit genes in the training set, multivariate Cox regression analysis and LASSO (shrinkage and selection method for linear regression) model were conducted to identify the prognostic value of these genes (Tibshirani, 1997). All the genes with significant *P*-values were screened to carry out multivariate Cox regression analysis to develop a prognostic signature to calculate the risk score of each patient (O'Quigley and Moreau, 1986).

To validate the robustness of the CCT subunit genes signature, the risk score of every HCC patient in the training, validation, and testing set was calculated. The patients were randomly separated into high- and low-risk score groups with the median scores as cut-off points. Meanwhile, the receiver operating characteristic curve (ROC) and Decision curve analysis (DCA) were performed to evaluate the prediction accuracy of the genes signature.

### Verifying the Expression of CCT4 and CCT6A

Normal liver cell line LO2 and human hepatic cancer cell line HepG2 were stored in our laboratory. Firstly, the total RNA was isolated form LO2 and HepG2 cells using TRIzol (Wanleibio Co. Ltd., Shanghai, China) and reverse transcribed into cDNA using the reverse transcriptase MMLV kit (Wanleibio Co. Ltd., Shanghai, China). Subsequently, the expression of CCT4, CCT6A, and CCT6B were detected by Real-Time Quantitative Polymerase Chain Reaction (RT-qPCR). Below the list of primers, β-Actin: forward primer 5′-GGCACCCAGCACAATGAA-3′ and reverse primer 5′-CGGACTCGTCATACTCCTGCT-3′, CCT6A: forward primer 5′-GCTAAAAGGAGAAATATGGAGAGG-3′ and reverse primer 5′-AATAATGTGACAGAACGAGGGT-3′, CCT4: forward primer 5′-GCTGGTTCTCACCCAAAAA-3′and reverse primer 5′-ATAGGCTCTCTCTTCTCGCA-3′, CCT6B: forward primer 5′- ACAGGTGAGCCAATGGTAGC-3′, and reverse primer 5′- CCAGGAGAATGTTGGTGGCA -3′.

### Statistical Analysis

All analyses were carried out using R statistical software (version 3.6, R Foundation for Statistical Computing, Vienna, Austria) with survival, clusterProfiler, ggplot2, and survROC package. Wilcoxon paired test was used to analyze the statistical difference in the expression of genes between tumor and non-tumor tissues. Survival analysis was performed between high- and low-risk groups using Kaplan-Meier analysis and two-sided log-rank test. The co-expressed relationships between the CCT subunit genes were computed using Pearson's test. Univariate and multivariate analysis of CCT subunit genes and clinical features were performed using Cox regression analysis. The nomogram was established in combination with gender, age, stage. The *P*-value < 0.05 was considered statistically significant.

## Results

### Patient Characteristics

Detailed characteristics of the 370 patients in the TCGA database, 232 in the ICGC database and 209 patients from GEO database were shown in Table 1.

**Table 1:** Patient characteristics.

**Clinical characteristics**		**TCGA**	**%**	**ICGC**	**%**	**GEO**	**%**
		370		232		209	
Survival status	Survival	244	65.95	189	81.47	130	62.2
	Death	126	34.05	43	18.53	79	37.8
Age	< =65years	232	62.70	90	38.79		
	>65 years	138	37.30	142	61.21		
Gender	Male	249	67.30	171	73.71		
	Female	121	32.70	61	26.29		
Histological grade	G1	55	14.86	NA			
	G2	177	47.84	NA			
	G3	121	32.70	NA			
	G4	12	3.24	NA			
Stage	I	171	46.22	36	15.52		
	II	85	22.97	106	15.69		
	III	85	22.97	71	30.60		
	IV	5	1.35	19	8.19		
T classification	T1	181	48.92	NA			
	T2	93	25.14	NA			
	T3	80	21.62	NA			
	T4	13	3.51	NA			
	TX	1	0.27	NA			
M classification	M0	266	71.89	NA			
	M1	4	1.08	NA			
	MX	100	27.03	NA			
N classification	N0	252	68.11	NA			
	N1	4	1.08	NA			
	NX	113	30.54	NA			
Prior malignancy	No	NA	NA	202	87.07		
	Yes	NA	NA	30	12.93		

*TCGA, The Cancer Genome Atlas; ICGC, International Cancer Genome Consortium, GEO, Gene Expression Omnibus*.

### CCT Subunit Gene Expression Levels

**Figure 1:**
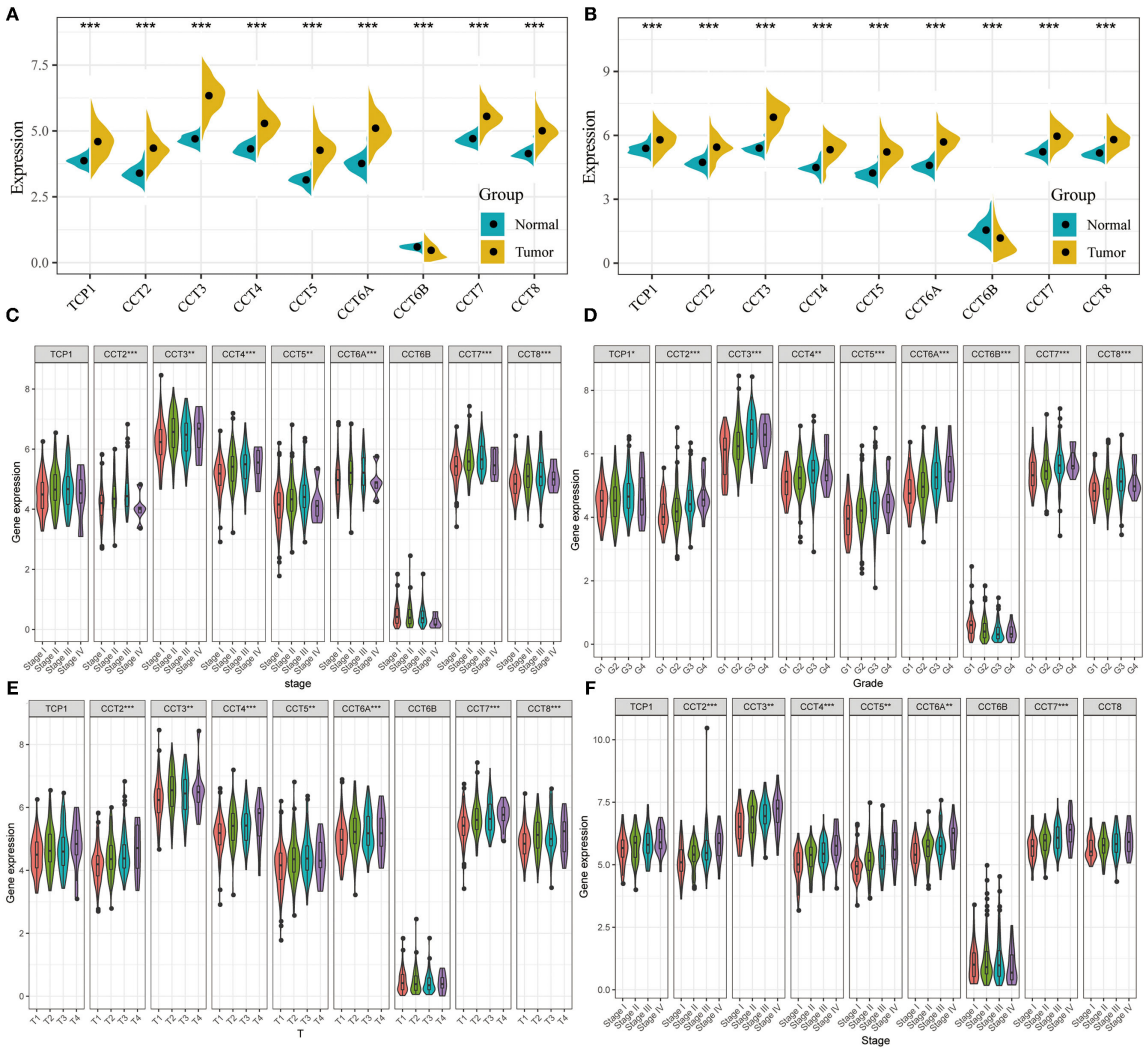
Comparison of CCT subunit genes expression in the TCGA and ICGC cohorts. CCT subunits (TCP1, CCT2, CCT3, CCT4, CCT5, CCT6A, CCT6B, CCT7, CCT8) gene expression levels were compared between tumor and non-tumor tissues in HCC patients from TCGA **(A)** and ICGC **(B)** cohorts. Comparison of CCT subunit gene expression levels with low and high stage **(C)**, grade **(D)** and T stage **(E)** in TCGA and ICGC **(F)** cohort. TCGA, The Cancer Genome Atlas; ICGC, International Cancer Genome Consortium. *P < 0.05, **P < 0.01, ***P < 0.001.

All nine members of the CCT subunit genes (TCP1, CCT2, CCT3, CCT4, CCT5, CCT6A, CCT6B, CCT7, CCT8) had their expression analyzed in TCGA (Figure 1A) and ICGC database (Figure 1B). The expression levels of TCP1, CCT2, CCT3, CCT4, CCT5, CCT6A, CCT7, and CCT8 were significantly higher in HCC tumor tissues than which in non-tumor tissues in the two databases (Figures 1A,B). However, the expression levels of CCT6B were decreased in tumor tissues compared with non-tumor tissues in the two databases (Figures 1A,B).

The expression levels of CCT2, CCT3, CCT4, CCT5, CCT6A, CCT7, and CCT8 were compared between patients with low and high stage HCC in the TCGA cohort (Figure 1C). It was conducted that the expression levels of TCP1, CCT2, CCT3, CCT4, CCT5, CCT6A, CCT7, and CCT8 were significantly higher in Grade 3 than which in Grade 1&2 in the TCGA cohorts. In contrast, the expression levels of CCT6B were significantly lower in Grade 3&4 than which in Grade 1&2 in the TCGA cohorts (Figure 1D). Meanwhile, the expression levels of CCT2, CCT3, CCT4, CCT5, CCT6A, CCT7, and CCT8 were increased significantly along with the T stage increased (Figure 1E). The same results were observed in the ICGC cohort (Figure 1F).

### Survival Analysis of CCT Subunit Genes

**Figure 2:**
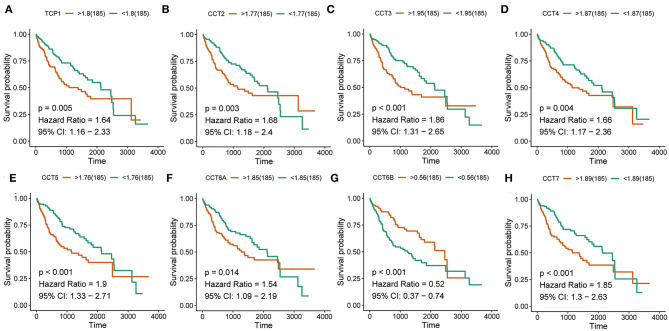
Survival analysis of HCC patients with respect to CCT subunit gene expression. Kaplan-Meier survival analysis was performed in HCC patients from the TCGA cohort. *P*-value was calculated using log-rank test and is provided at the top left of each figure.

**Figure 3:**
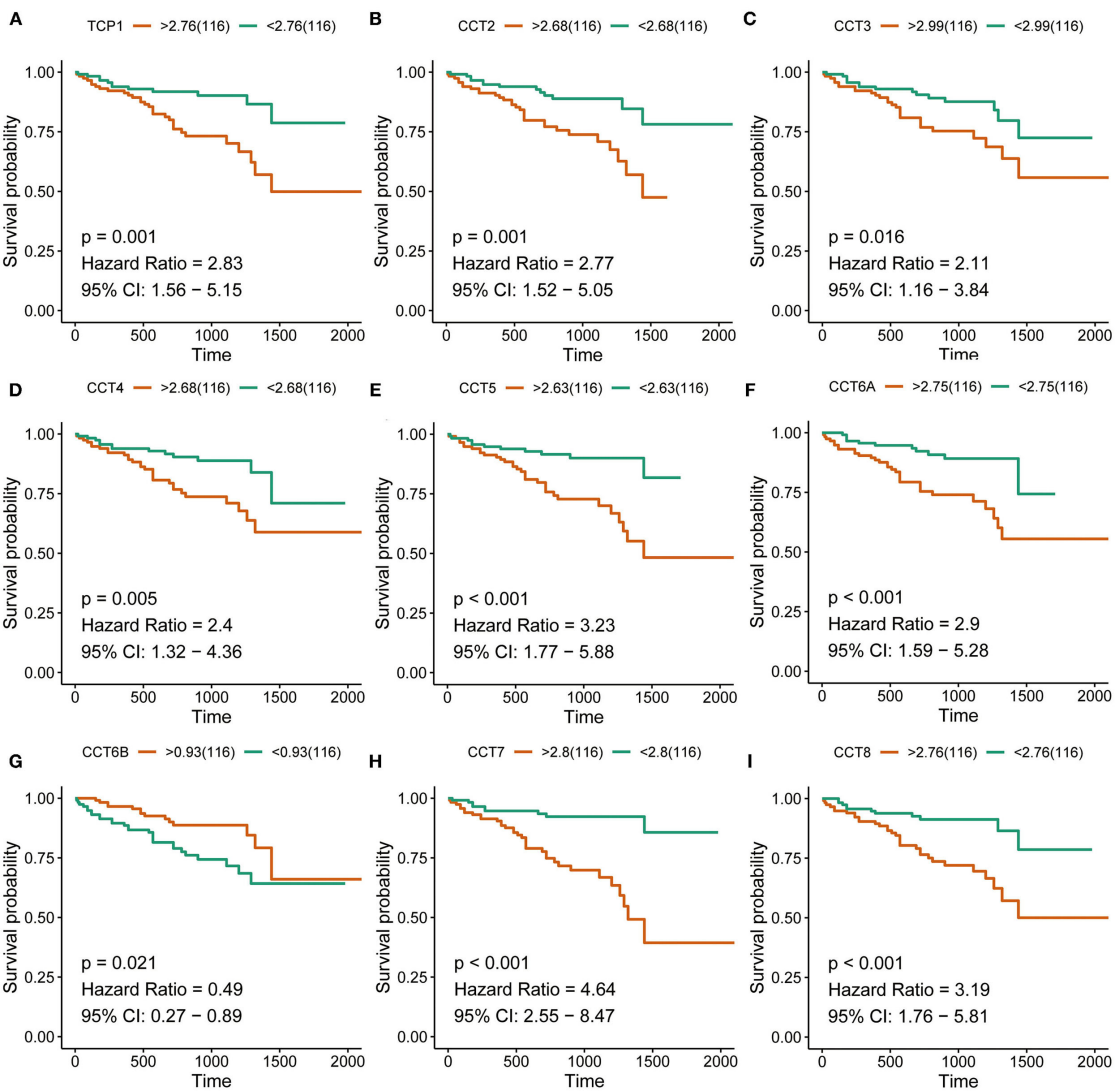
Survival analysis of HCC patients with respect to CCT subunit gene expression. Kaplan-Meier survival analysis was performed in HCC patients from the ICGC cohort. *P*-value was calculated using log-rank test and is provided at the top left of each figure.

Interestingly, high expression of TCP1, CCT2, CCT3, CCT4, CCT5, CCT6A, CCT7, and CCT8 was significantly associated with poorer prognosis for HCC patients from the TCGA and ICGC cohorts (all *P* < 0.01; Figures 2A–H, Figures 3A–I). High expression of CCT6B was significantly associated with better overall survival in the TCGA and ICGC cohorts (all *P* < 0.01; Figures 2G, 3G).

### Correlation Analysis of the Expression Levels Among CCT Subunit Genes

**Figure 4:**
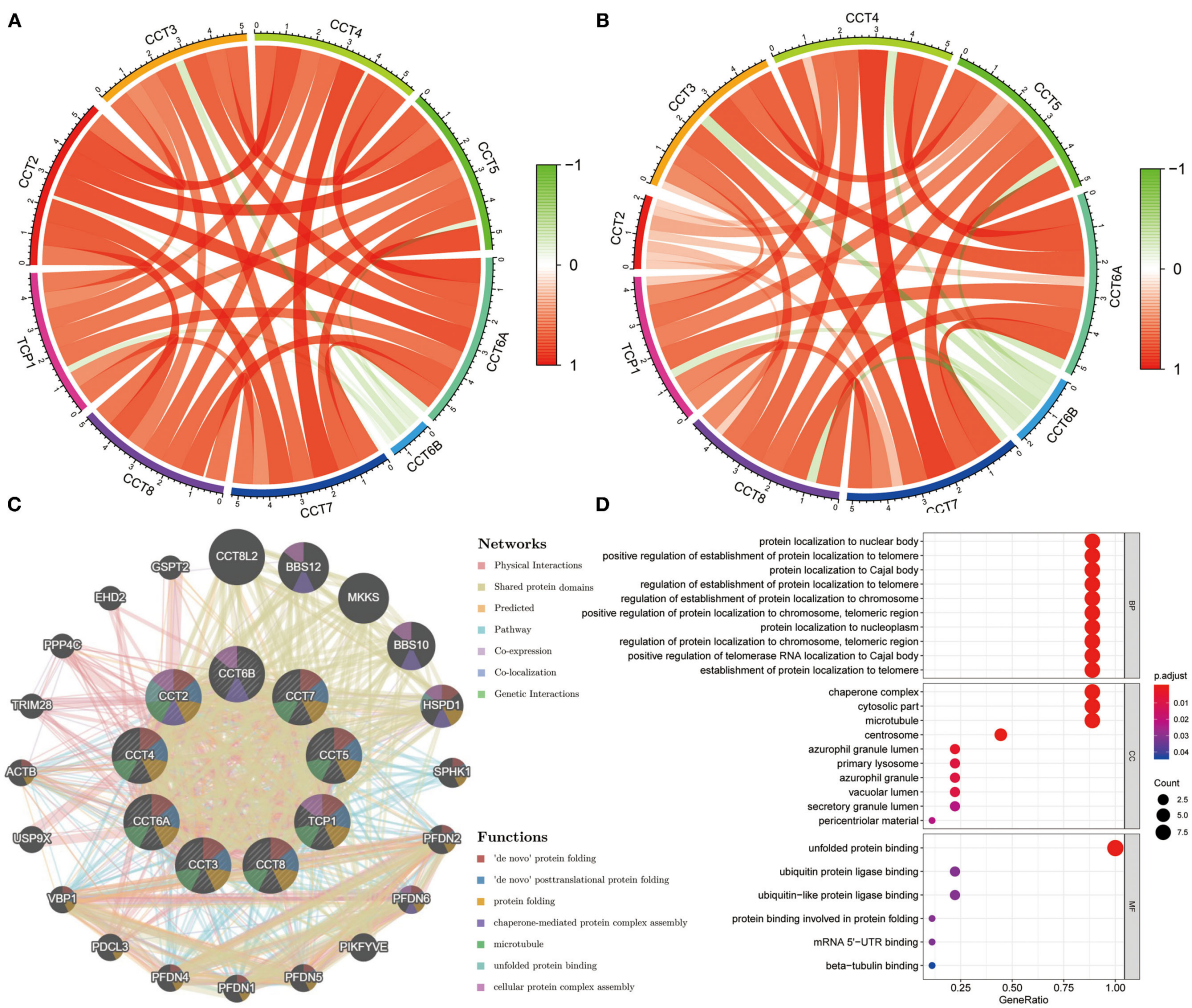
Expression correlation among CCT subunit genes and protein-protein interaction (PPI) network analysis of CCT subunit proteins. **(A)** Expression correlation among CCT subunit genes in the TCGA cohort. **(B)** Expression correlation among CCT subunit genes in the ICGC cohort. **(C)** PPI network of CCT subunit proteins in the GeneMANIA database. **(D)** Gene ontology and pathway enrichment analysis of CCT subunit proteins.

The Pearson correlation coefficients of all CCT subunit genes were identified. In the TCGA and ICGC database, each of these nine genes was positively and significantly associated with the other eight members (all *P* < 0.05; Figures 4A,B). Meanwhile, protein-protein interaction networks were constructed using the GeneMANIA database, which showed that the nine CCT subunit proteins were also associated with others (Figure 4C).

Gene Ontology analysis of CCT subunit genes revealed enrichment of biological pathways linked to unfolded protein binding, protein localization to nuclear body, positive regulation of establishment of protein localization to telomere and protein localization to Cajal body and so on (Figure 4D).

### Univariate and Multivariate Analysis of CCT Subunit Genes

To explore the effect of nine prognostic CCT subunit genes and clinical features, the prognostic-related clinical characteristics in the TCGA database of age, gender, stage, TNM, and CCT subunit genes were analyzed using univariate analysis respectively, which showed that TCP1, CCT2, CCT3, CCT4, CCT5, CCT6A, CCT6B, CCT7, CCT8, and stage exhibited significant relationships with prognosis of HCC (all *P* < 0.05; Supplementary Table 1). The results of the univariate analysis were consistent in the ICGC cohort (all *P* < 0.05; Supplementary Table 2).

Furthermore, we also conducted multivariate Cox regression analysis with each CCT subunit gene and clinical characteristics in the two cohorts (Supplementary Figures 1, 2). These results have shown that each CCT subunit gene was significantly associated with the survival in the TCGA cohort (all *P* < 0.01; Supplementary Figure 1). In the ICGC cohort, CCT subunit genes were significantly associated with the overall survival (all *P* < 0.01; Supplementary Figure 2) except CCT6B, which showed approaching significance (*P* = 0.06).

### CCT Subunit Genes Expression Correlated With Immune Cell Infiltration

**Figure 5:**
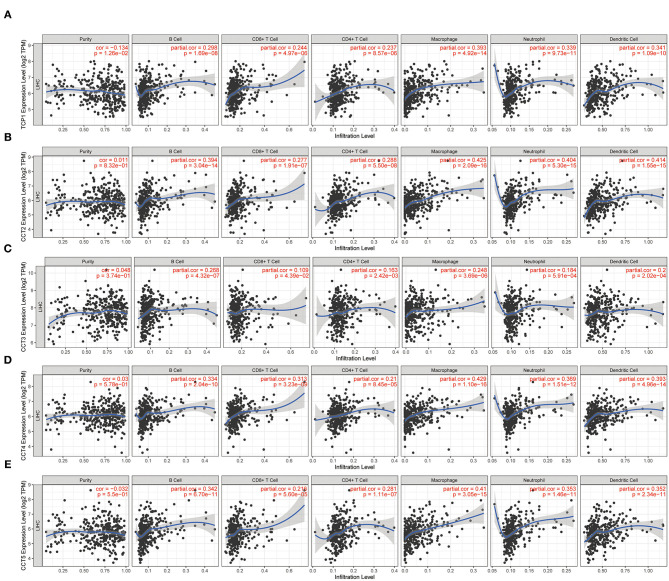
Correlation of immune cell infiltration with TCP1 **(A)**, CCT2 **(B)**, CCT3 **(C)**, CCT4 **(D)**, CCT5 **(E)**.

**Figure 6:**
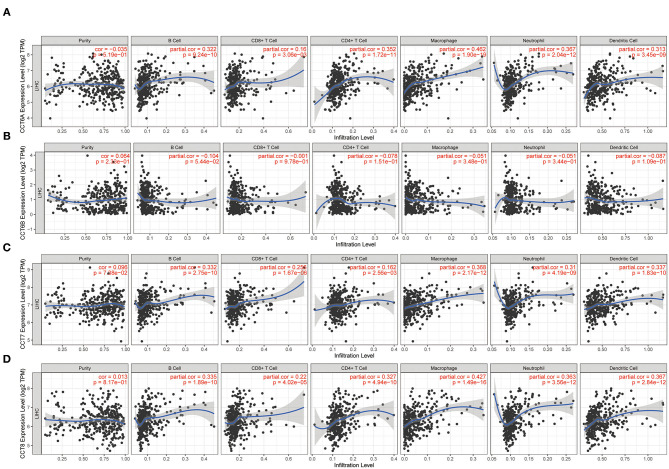
Correlation of immune cell infiltration with CCT6A **(A)**, CCT6B **(B)**, CCT7 **(C)**, CCT8 **(D)**.

**Figure 7:**
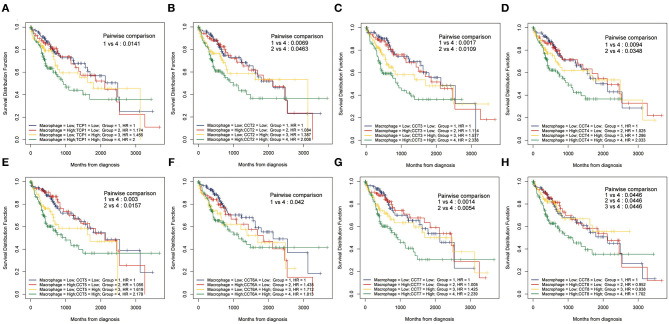
Kaplan-Meier analysis using combinations CCT subunit genes and Macrophage score. Overall survival analysis of combinations Macrophage score and TCP1 **(A)**, CCT2 **(B)**, CCT3 **(C)**, CCT4 **(D)**, CCT5 **(E)**, CCT6A **(F)**, CCT7 **(G)**, and CCT8 **(H)**.

While the analyses results revealed that CCT subunit genes were independent predictors of HCC patients, their potential function remain to be discovered. Growing studies suggest that tumor-infiltrating immune cells play a critical role in tumor development and progression. However, it is unclear whether CCT subunit genes can influence the recruitment of immune cells. As such, we analyzed the relationship between CCT subunit genes expression and immune cell infiltration using TIMER database. The results indicated that CCT subunit genes presented a positive correlation with B cell, CD4+ T cell, CD8+ T cell, Macrophage, Neutrophil, and Dendritic cell (Figures 5, 6). Notably, TCP1, and CCT2/3/4/5/6A/7/8 showed a strong correlation with Macrophage (Figures 5, 6). As such, we further explored the correlation of TCP1, and CCT2/3/4/5/6A/7/8 aberrant expression associated Macrophage with overall survival. Firstly, we split the HCC patients into four group based on the expression of CCT subunit genes and Macrophage score. Then, we observed that the group with high expression of TCP1, or CCT2/3/4/5/6A/7/8 and high Macrophage score significantly presented worst overall survival compare to others group (Figure 7).

### Construction of Transcription Factor Regulatory Networks

**Figure 8:**
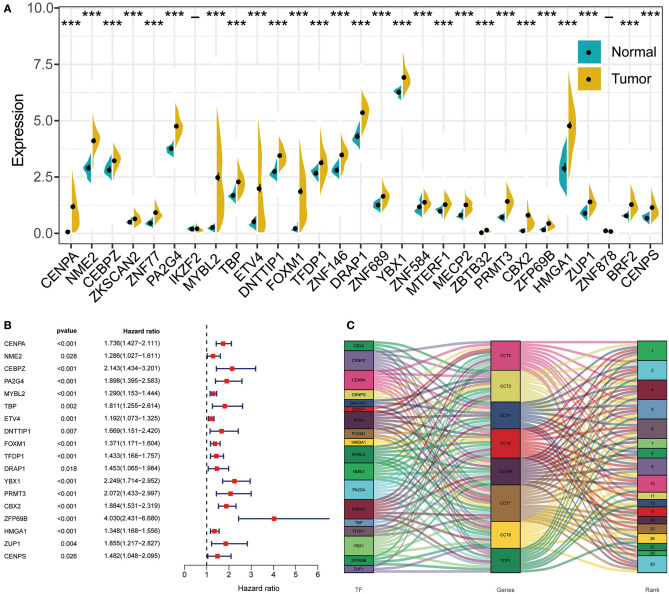
The regulation network between transcription factors and CCT subunit genes. **(A)** Differentially expressed transcription factors in HCC tissues and normal tissue. *P < 0.05, **P < 0.01, ***P < 0.001. **(B)** Univariate COX survival analysis of differentially expressed transcriptional factors in HCC. **(C)** Regulation network of CCT subunit genes and survival-related transcription factors.

Transcription factor can drive the expression of many target genes, and serve important functions in cancer occurrence and metastasis. Thus, we enriched transcription factors potentially involved in the regulation of CCT subunit genes. Next, we analyzed the expression of the top 30 transcription factors in HCC and adjacent noncancerous tissues (Figure 8A). Subsequently, we obtained 18 overall survival-related transcription factors by univariate Cox regression (Figure 8B). Finally, we built detailed regulatory networks linking transcription factor to CCT subunit genes (Figure 8C). The results showed that PA2G4, CEBPZ, PRMT3, YBX1, and CENPA may play a more important role in participating in TCP1, CCT2/3/4/5/6A/7/8 transcription.

### The Prognostic Signature Based on the Expression of CCT Subunit Genes

To develop a prediction signature, CCT subunit genes (TCP1, CCT2, CCT3, CCT4, CCT5, CCT6A, CCT6B, CCT7, and CCT8) in the training set were subjected to the LASSO model (Supplementary Figure 3A). As a result, CCT4, CCT6A, CCT6B, and CCT8 were significantly correlated to the prognostic of HCC patients. These genes were selected for next-step model construction by multivariable Cox proportional hazard regression (Supplementary Figure 3B). In total, the signature comprised of 3 optimal genes, including CCT4, CCT6A, and CCT6B. CCT4 and CCT6A had positive coefficients, demonstrating that the expression of them level was observed in patients with good prognostic. The negative coefficients for CCT6B represented that the higher expression level of CCT6B was observed in patients with poor survival. Thus, the risk score signature was constructed: risk score = (0.013805 × expression level of CCT4) + (0.015096 × expression level of CCT6A) + (-0.74942 × expression level of CCT6B). By calculating the risk score of every patient in the training, validation and testing set, the patients were divided into low- and high-risk groups using the median score.

**Figure 9:**
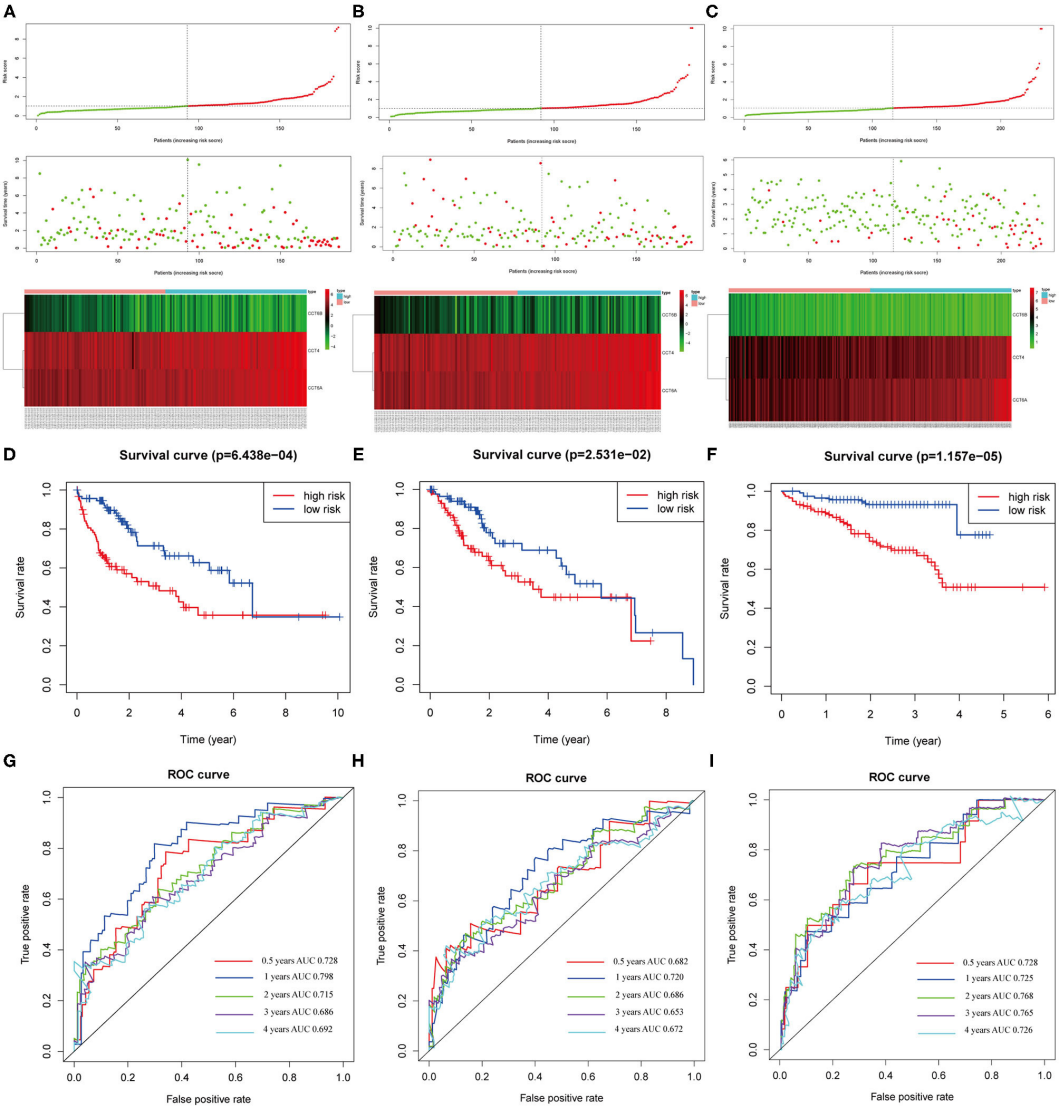
Correlation between the 3-gene signature and the overall survival of patients in the training, validation and testing set. The distribution of risk scores, survival time and gene expression levels in the training **(A)**, validation **(B)**, and testing set **(C)**. Kaplan–Meier curves for overall survival of the low- and high-risk groups in the training **(D)**, validation **(E)** and testing set **(F)**. ROC curves for the 0.5-, 1-, 2-, 3-, 4-years survival prediction by the 3-gene signature in the training **(G)**, validation **(H)**, and testing set **(I)**.

The distribution of patient risk scores in the training, validation, and testing set, the survival status and CCT subunit genes expression of HCC patients are shown in Figure 9 and ranked according to the 3-gene prognostic signature. Figure 9A upper figure showed patients sorted based on risk score, with green indicating patients with a risk score below the median and red indicating those with a risk score above the median. Figure 9A middle and below showed that patients who had high-risk scores were inclined to express hazardous genes, while patients with low-risk scores were inclined to express protective genes. The death rate of patients with high-risk scores is higher than in those with low-risk scores. Additionally, we observed similar results in the internal and external validation set (Figures 9B,C and Supplementary Figure 4).

The Kaplan-Meier analysis suggested that patients in the high-risk group had a worse prognosis than patients in the low-risk group in the training, validation and testing set (all *P* < 0.05; Figures 9D–F). The risk score signature showed a great survival prediction in HCC patients. The 0.5-, 1-, 2-, 3-, 4- years area under ROC curves (AUC) of the 3-gene prognostic signature in the training, validation and testing set were all more than 0.65 and 1- year area under ROC curves (AUC) of the 3-gene prognostic signature were all over 0.72 which showed good predictive ability (Figures 9G–I).

**Figure 10:**
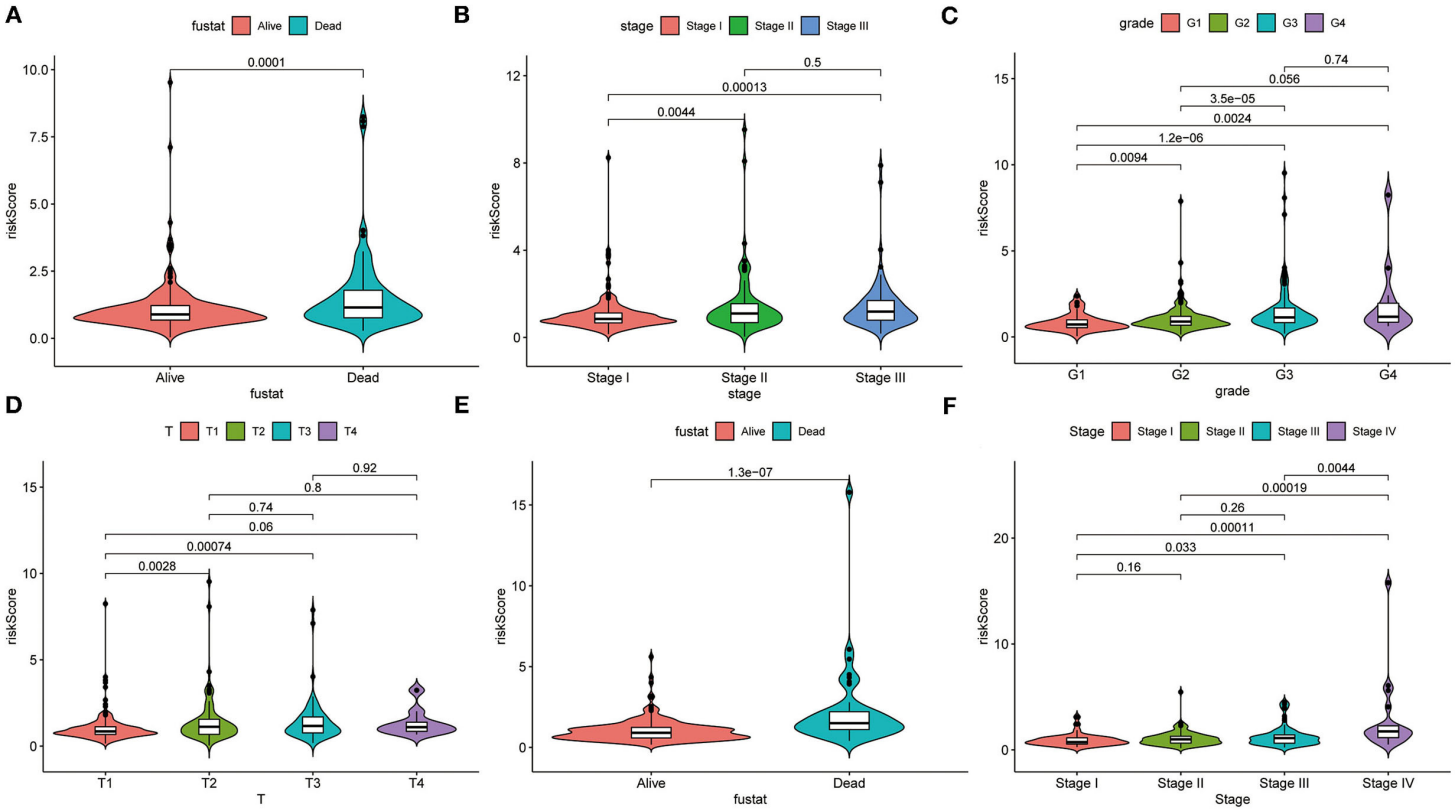
Correlation analysis between clinical feature and risk score. The distribution of risk score in different survival statue **(A)**, clinical stage **(B)**, histologic Grade **(C)**, T stage **(D)** based on TCGA cohort. The distribution of risk score in different survival statue **(E)**, clinical stage **(F)**.

The heatmap shows clinical characteristics and the expression of CCT6B, CCT4 and CCT6A in low- and high-risk patients in the training, validation and testing set (Supplementary Figure 5). We found that significant difference between the low- and high-risk patients with respect to survival outcome (*P* < 0.05) in the training set, grade (*P* < 0.001) in the validation, stage (*P* < 0.01), and survival outcome (*P* < 0.001) in the testing set. Moreover, we also checked the relationship between risk score and clinical feature in TCGA (training and validation set) and ICGC (testing set) cohorts. The results indicated that HCC patients who died had a higher risk score compared to survival patients in TCGA cohort (Figure 10A). Meanwhile, we found that the risk score was significantly increased with TNM Stage, Grade stage, and T stage (Figures 10B–D). Furthermore, we observed the same trend in ICGC cohort (Figures 10E,F).

### Univariate and Multivariate Analysis of 3-Gene Prognostic Signature

**Figure 11:**
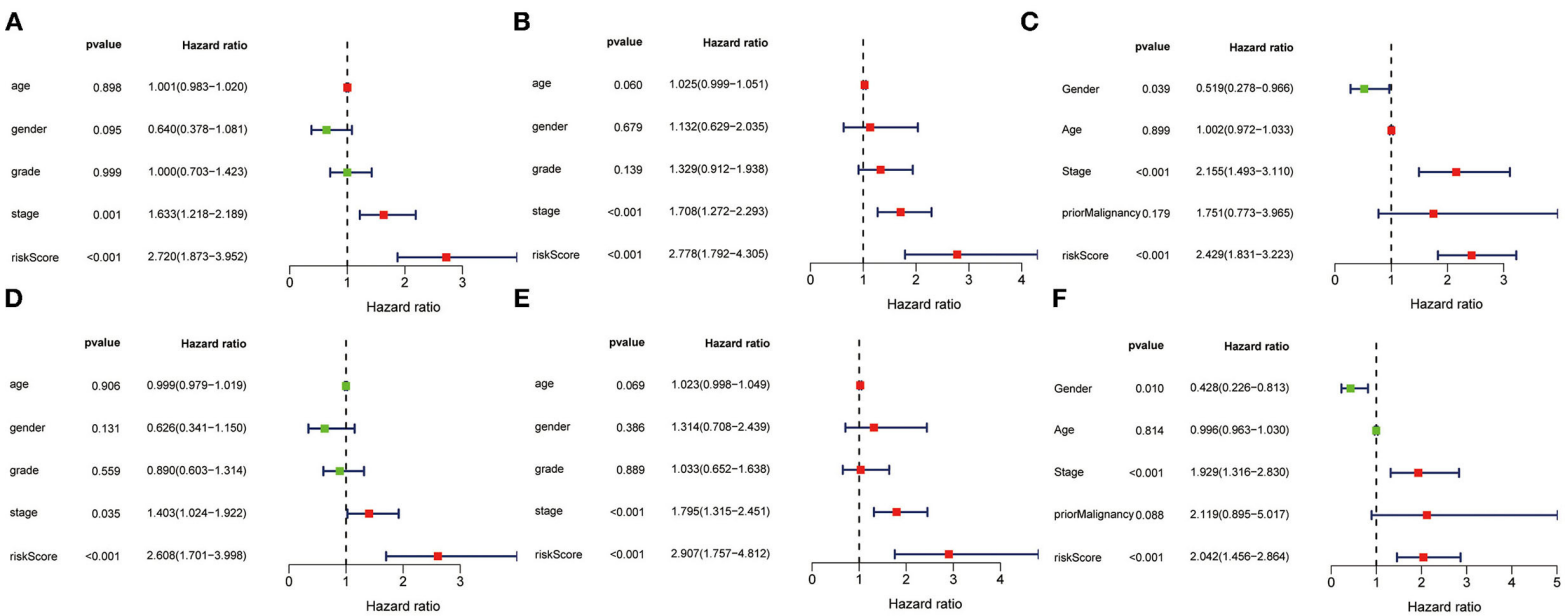
Univariate and multivariate analysis of 3-gene signature and clinical characteristics in the training, validation and testing set. Univariate analysis of 3-gene signature and clinical characteristics in the training **(A)**, validation **(B)** and testing set **(C)**. Multivariate analysis of 3-gene signature and clinical characteristics in the training **(D)**, validation **(E)** and testing set **(F)**.

The univariate and multivariate Cox regression models of 3-gene prognostic signature were performed in the training, validation and testing set. In the training and validation set, age, gender, grade, stage and risk score calculated from the 3-gene signature were included (Figures 11A,B for univariate Cox regression; Figures 11D,E for multivariate Cox regression analysis). In the testing set, gender, age, stage, prior malignancy, and risk score calculated from the 3-gene signature were included (Figure 11C for univariate regression and Figure 11F for multivariate Cox regression). Univariate and multivariate Cox regression analysis indicated that stage and risk score calculated from the three-gene signature were independent prognostic factors for overall survival (*P* < 0.05).

### Constructing and Assessing a Nomogram

**Figure 12:**
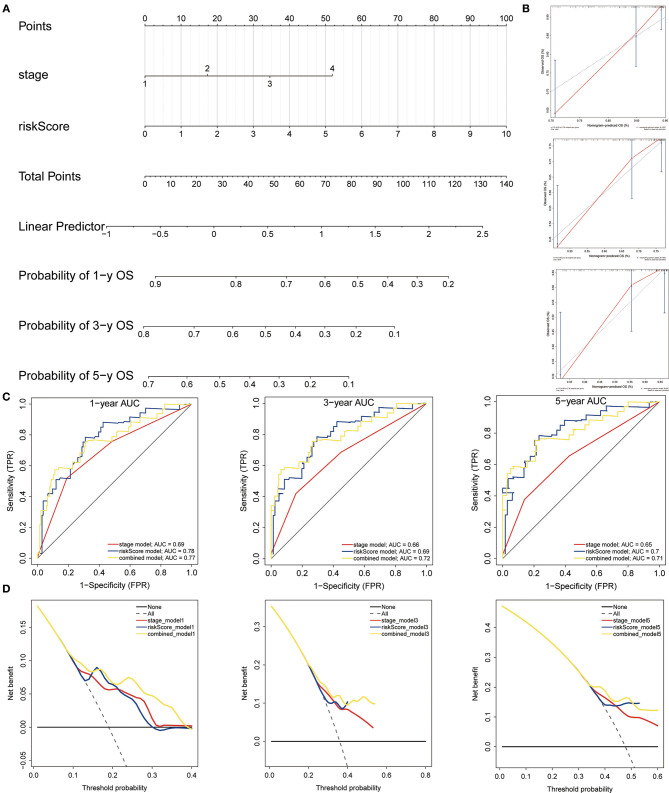
Construction and evaluation of the nomogram. **(A)** The nomogram of 3-gene prognostic signature in the TCGA cohort. **(B)** Calibration plots of the nomogram for estimation of survival at 1-, 3-, 5- year. **(C)** ROC curves showed the predictive efficiency of the nomogram. **(D)** Relations between net benefit and threshold probability at 1-year, 3-year, and 5-year survival predictions.

The nomogram was built to predict 1-, 3- and 5-year survival using the 3-gene signature and other clinical features in the TCGA cohort (including stage and risk score calculated by 3-gene signature) (Figure 12A). Meanwhile, calibration curves exhibited that the nomogram performed relatively well calibrated, which closed to the best prediction (Figure 12B). Subsequently, the ROC cure was performed to evaluate the predictive efficiency of the nomogram. The AUC of combined nomogram were 0.77, 0.72, and 0.71 for predicting 1-, 3-, 5-years survival, respectively (Figure 12C). At the same time, the combined model presented greater net benefit compared to risk score or stage alone (Figure 12D).

### The Expression of CCT4, CCT6B and CCT6A *in vitro*

**Figure 13:**
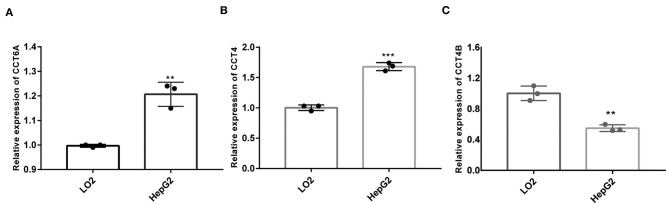
Comparison of mRNA expression between HepG2 and LO2 on CCT4 and CCT6A. **(A)** CCT6A mRNA expressions in two cell lines. **(B)** CCT4 mRNA expressions in two cell lines. **(C)** CCT6B mRNA expression in two cell lines. **P < 0.01; ***P < 0.001.

In order to further validate the expression of CCT4, CCT6B, and CCT6A, we investigated the relative expression of its transcripts in HCC and LO2 cell lines by quantitative rt-PCR. The results showed that the expression level of CCT4, CCT6B, and CCT6A were significantly upregulated in HCC cells HepG2 as compared to normal liver cells LO2 (Figure 13).

## Discussion

Scientists have confirmed that CCT played a key role in cytosolic protein folding and assembly (Vallin and Grantham, 2019). Thus, it is associated with HCC proliferation, progression, and invasion (Huang et al., 2014; Cui et al., 2015; Zhang et al., 2016; Gao et al., 2019). With the advancement of molecular profiling, molecular markers were identified to improve the understanding of the molecular heterogeneity of HCC and facilitate individualized treatment such as protein-coding genes. Recently, some researchers identified a novel HSP signature including three CCT genes for the prognosis of breast cancer patients (Klimczak et al., 2019). The prognostic value of CCT subunit genes for HCC has not been systematically investigated yet.

In the present study, we identified nine CCT subunit genes (TCP1, CCT2, CCT3, CCT4, CCT5, CCT6A, CCT6B, CCT7, CCT8) as independent prognostic factors for survival in HCC patients (Klimczak et al., 2019). Meanwhile, we found that CCT subunit genes aberrant expression was associated with immune cell infiltration, especially with Macrophage. And we also discovered that HCC patients with high expression of CCT subunit genes and high Macrophage score had unfavorable prognosis. This, maybe, is due to Macrophage cell mainly exists as M2 type in HCC tumor immune microenvironment. Some of the genes were reported as cancer-related genes, such as CCT2 (Guest et al., 2015), CCT3 (Cui et al., 2015), CCT4 (Yu et al., 2016), CCT6A (Huang et al., 2019), CCT7 (Gao et al., 2019), CCT8 (Huang et al., 2014). After the multivariate Cox proportional hazards regression and LASSO model, we developed and validated a novel three-gene signature (including CCT4, CCT6A, CCT6B) which was significantly correlated with survival in HCC patients. Based on the univariate and multivariate analysis, we established a nomogram combining the 3-gene signature. Henceforth, it is necessary to conduct a further study to understand the mechanisms underlying the signature and nomogram. These results were consistent in the training, validation and testing set, indicating the robustness of the prognostic signature.

The genes in our signature have already been reported. Several researches have revealed that CCT4 expression level was associated with the prognostic of glioblastoma multiforme (Yu et al., 2016) and ovarian cancer (Wada et al., 2009). CCT6A expression was shown to be increased in 10 human tumor cell lines and associated with poor survival in lung cancer (Ying et al., 2017), breast cancer (Huang et al., 2019), and glioblastoma (Hallal et al., 2019). As far as we know, few researchers have focused on the function of CCT6B in tumors. It was only investigated that CCT6B could be a therapeutic target of joint contracture (Yi et al., 2019) and sperm quality (Agarwal et al., 2016). In the TCGA cohort, we found the expression level of CCT6B was decreased in tumors when compared to normal tissues. Besides, high expression of CCT6B was related to the negative prognostic of HCC patients. Our results suggested that all three genes may serve as key functional genes, playing a crucial role in the HCC, but the molecular mechanisms have not yet been elucidated.

Nevertheless, several limitations are presented in this research. First, the number of HCC patients for screening CCT subunit genes was small. Second, we established the gene signature based on the expression levels of CCT subunit genes, without considering the mutation and methylation of genes or microRNAs, long noncoding RNAs that are associated with origin and progression of HCC. Third, it lacks experiment research to confirm the results. Last but not least, the 3-gene prognostic signature should be applied in the larger population of HCC patients from diverse backgrounds.

In conclusion, we developed and validated a 3-gene prognostic signature and nomogram comprised of three CCT associated genes. This signature could provide promising evidence to survival prediction and novel biomarkers to targeted therapy for HCC.

## Data Availability Statement

The original contributions presented in the study are included in the article/Supplementary Material, further inquiries can be directed to the corresponding author/s.

## Author Contributions

HZ, WL, and JL conceived and designed the study. JL, WL, and HZ collected and analyzed the data. HZ, WL, and JL wrote the manuscript. All authors read and approved the final manuscript and agree to be accountable for all aspects of the research in ensuring that the accuracy or integrity of any part of the work are appropriately investigated and resolved.

## Conflict of Interest

The authors declare that the research was conducted in the absence of any commercial or financial relationships that could be construed as a potential conflict of interest.

## Supplementary Material

Supplementary Figure 1 Multivariate analysis of CCT subunit genes and clinical characteristics in the TCGA cohort.

Supplementary Figure 2 Multivariate analysis of CCT subunit genes and clinical characteristics in the ICGC cohort.

Supplementary Figure 3 Construction of 3-genes signature. **(A)** The coefficients obtained from the LASSO algorithm. **(B)** Stepwise multivariate Cox regression was applied to built final model.

Supplementary Figure 4 Validation of 3-gene signature in GSE14520 cohort. **(A)** The distribution of risk scores, survival time and gene expression levels in GSE14520 cohort. **(B)** overall survival difference between high-risk and low-risk group.

Supplementary Figure 5 Relationship between risk score, clinical characteristics and expression of CCT6B, CCT4, and CCT6A. The heatmap shows clinical characteristics and the expression levels of CCT6B, CCT4, and CCT6A in the low- and high- group of HCC patients in training **(A)**, validation **(B)** and testing **(C)** set. ^*^*P* < 0.05, ^**^*P* < 0.01 and ^***^*P* < 0.001.

Supplementary Table 1 Univariate analysis of CCT subunit genes and clinical characteristics in the TCGA cohort.

Supplementary Table 2 Univariate analysis of CCT subunit genes and clinical characteristics in the ICGC cohort.
