# Supplementary figures and images for: Prognostic Power of a Chaperonin Containing TCP-1 Subunit Genes Panel for Hepatocellular Carcinoma
Source: Front Genet. 2021 Apr 8;12:668871. doi: 10.3389/fgene.2021.668871 (PMC8061729; doi:10.3389/fgene.2021.668871)

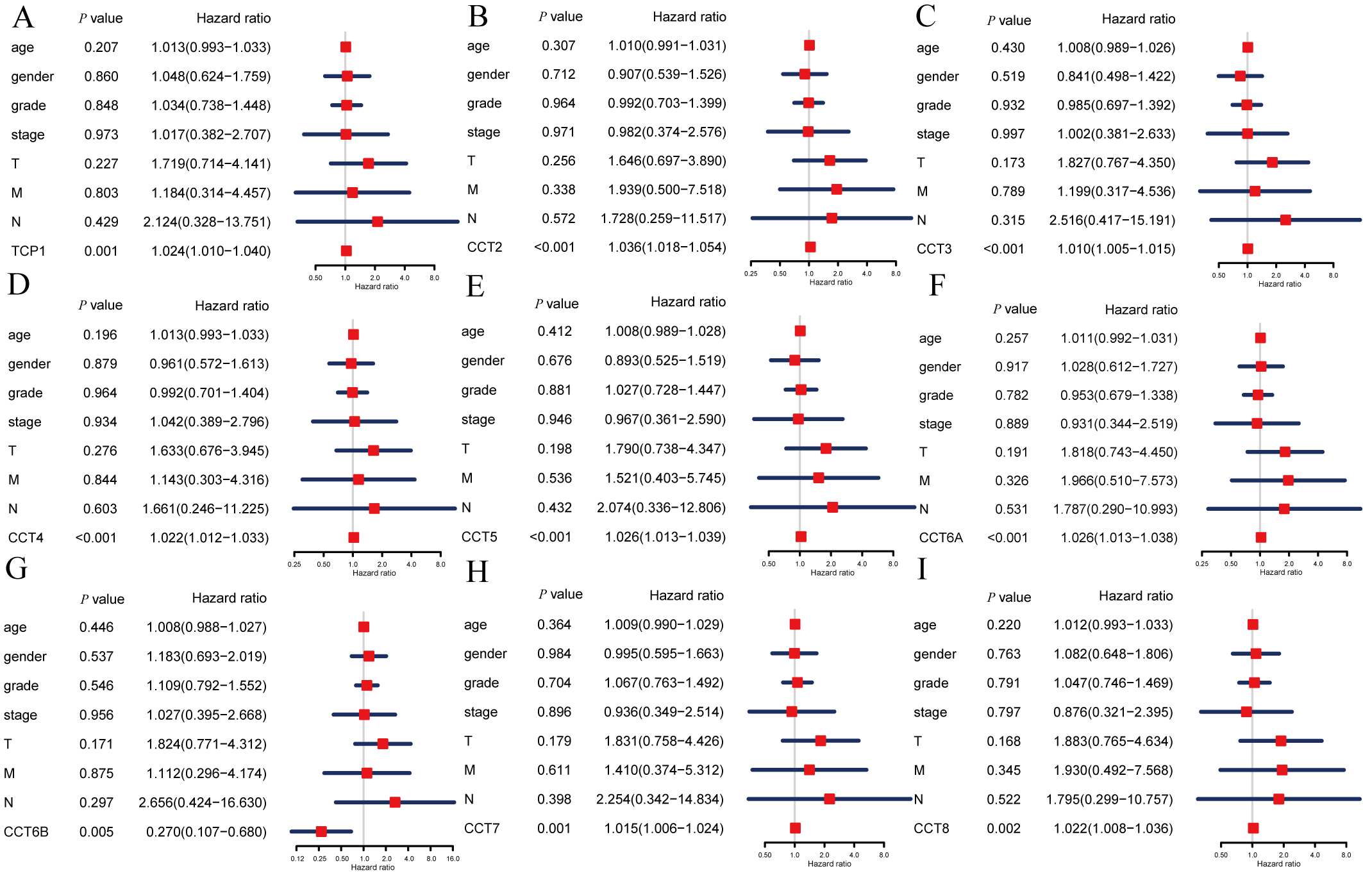

Supplement: Supplementary Figure 1 — Multivariate analysis of CCT subunit genes and clinical characteristics in the TCGA cohort. [file Image_1.TIF]

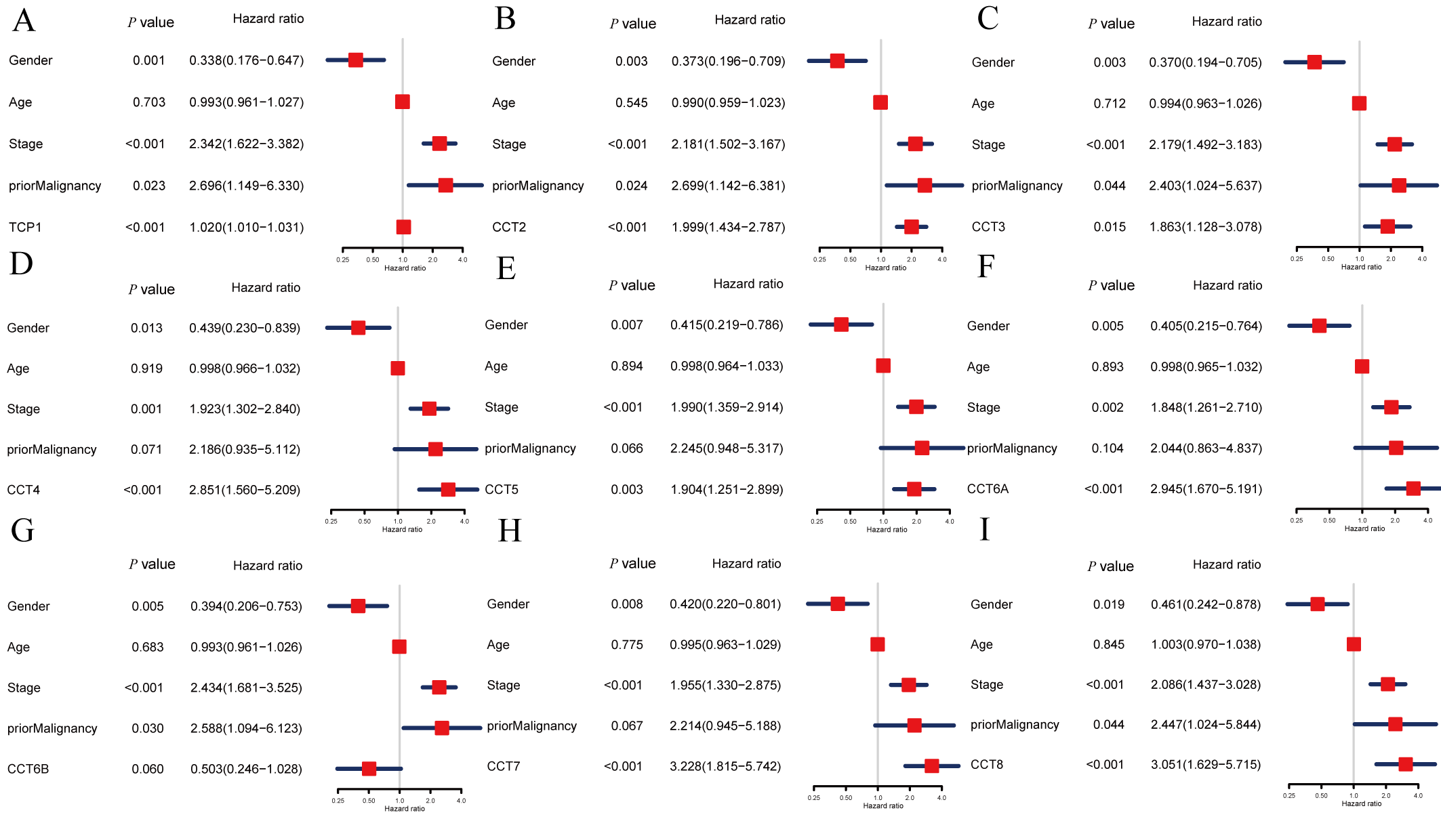

Supplement: Supplementary Figure 2 — Multivariate analysis of CCT subunit genes and clinical characteristics in the ICGC cohort. [file Image_2.TIF]

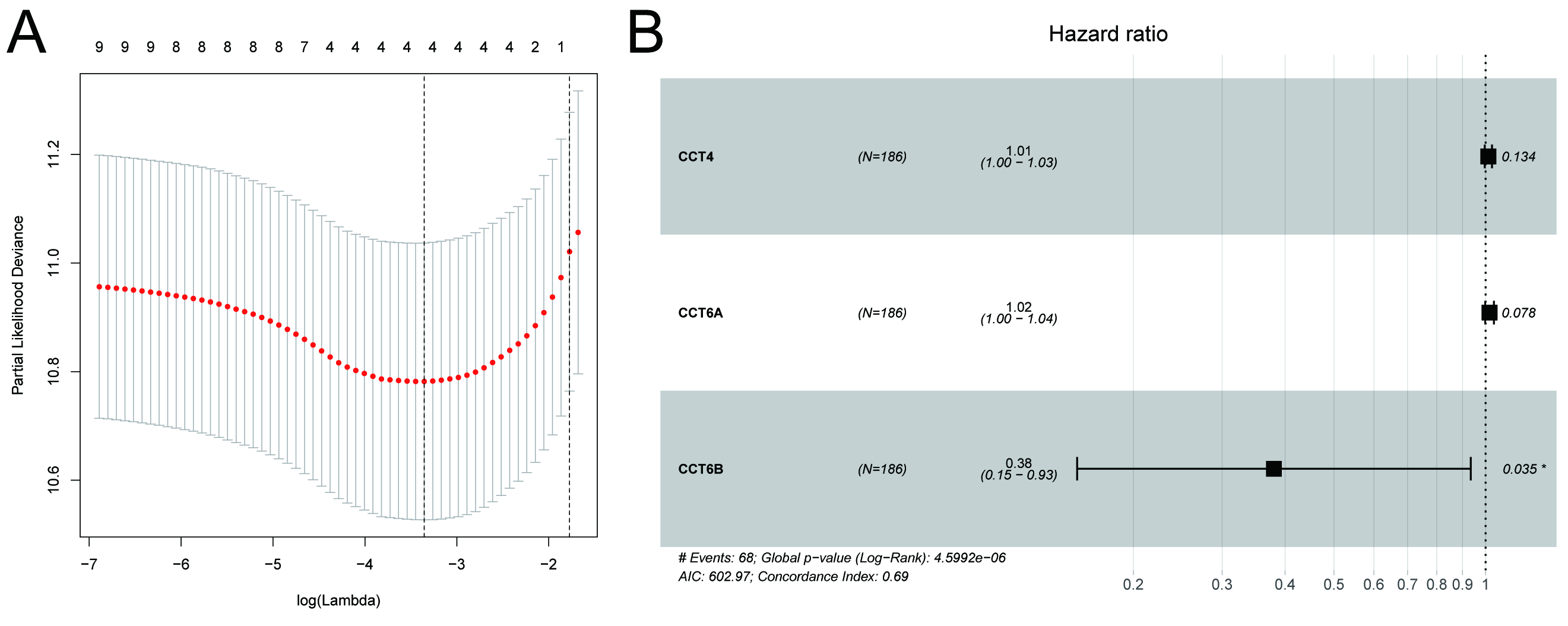

Supplement: Supplementary Figure 3 — Construction of 3-genes signature. (A) The coefficients obtained from the LASSO algorithm. (B) Stepwise multivariate Cox regression was applied to built final model. [file Image_3.TIF]

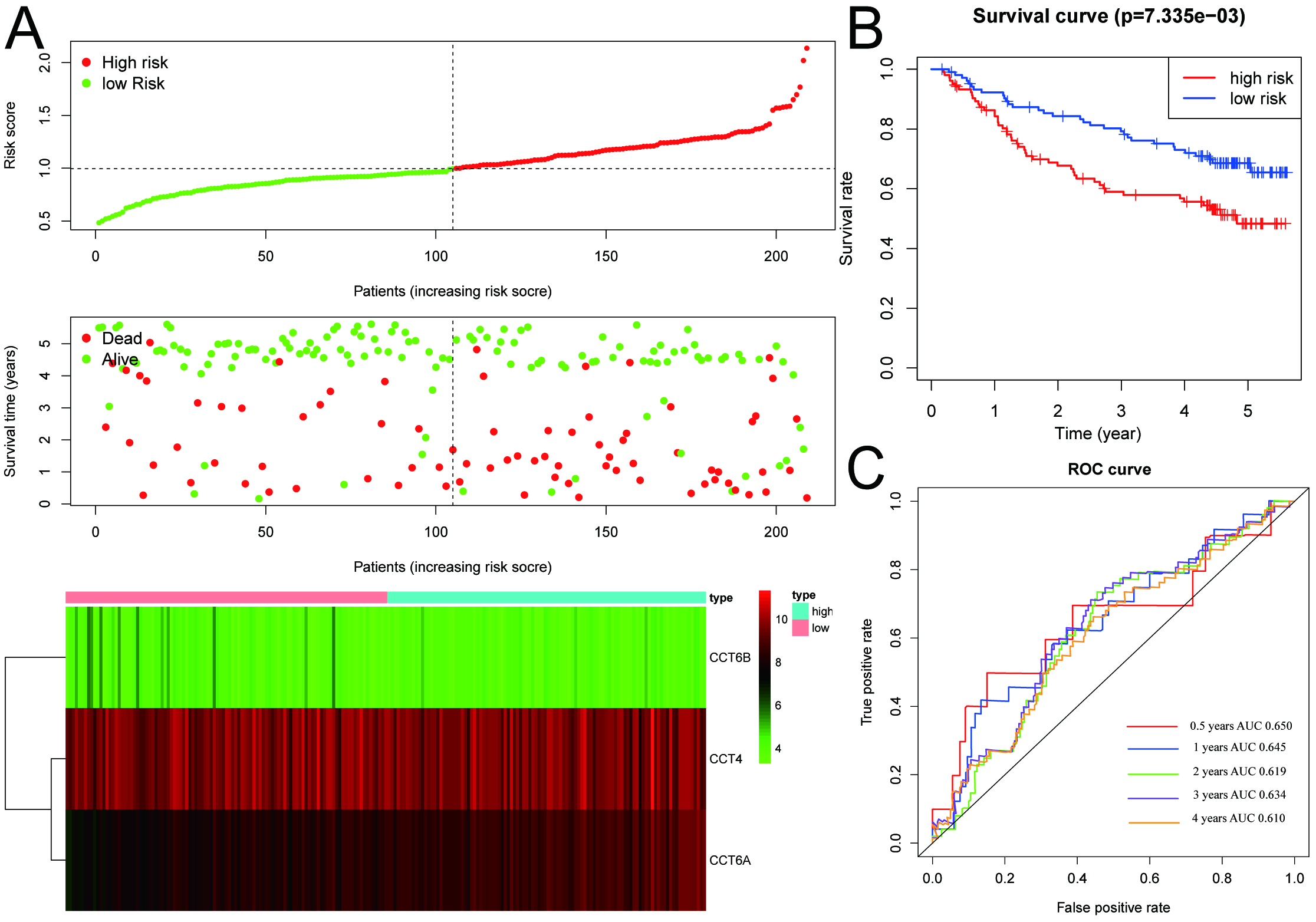

Supplement: Supplementary Figure 4 — Validation of 3-gene signature in GSE14520 cohort. (A) The distribution of risk scores, survival time and gene expression levels in GSE14520 cohort. (B) overall survival difference between high-risk and low-risk group. [file Image_4.TIF]

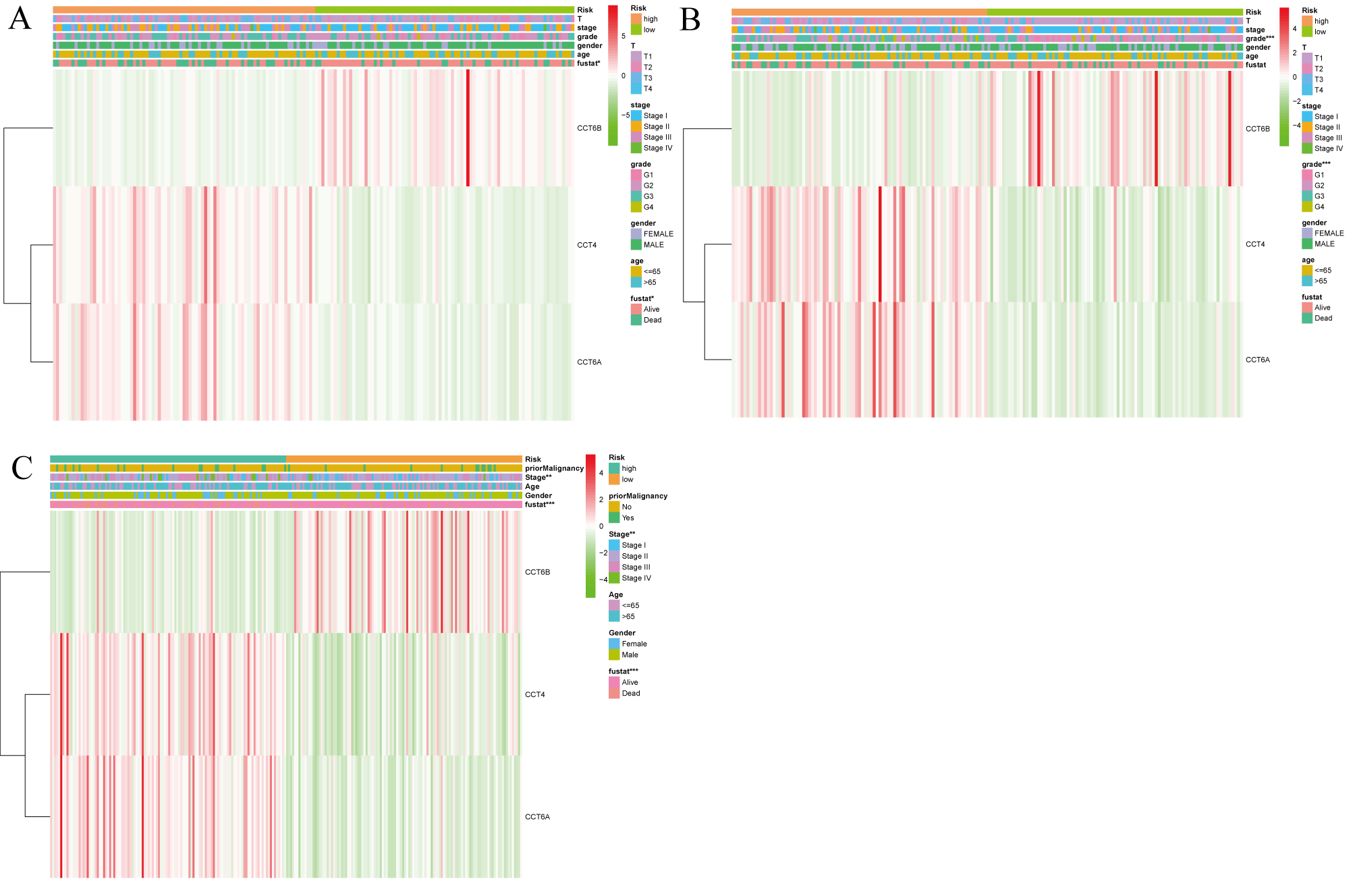

Supplement: Supplementary Figure 5 — Relationship between risk score, clinical characteristics and expression of CCT6B, CCT4, and CCT6A. The heatmap shows clinical characteristics and the expression levels of CCT6B, CCT4, and CCT6A in the low- and high- group of HCC patients in training (A), validation (B) and testing (C) set. *P < 0.05, **P < 0.01 and ***P < 0.001. [file Image_5.TIF]
