# Supplementary material for: Prognostic Power of a Chaperonin Containing TCP-1 Subunit Genes Panel for Hepatocellular Carcinoma
Source: Front Genet. 2021 Apr 8;12:668871. doi: 10.3389/fgene.2021.668871 (PMC8061729; doi:10.3389/fgene.2021.668871)
Supplement: Supplementary Table 1 — Univariate analysis of CCT subunit genes and clinical characteristics in the TCGA cohort. [file Data_Sheet_1.doc]

Table S1 Univariate analysis of CCT subunit genes and clinical characteristics in the TCGA cohort.

| **Variables** | **HR** | **95% CI for HR** | | ***P***-value |
| --- | --- | --- | --- | --- |
|  |  | **Lower** | **Upper** |  |
| Age | 1.005006 | 0.986857 | 1.02349 | 0.591219 |
| Gender | 0.780125 | 0.487182 | 1.249215 | 0.301298 |
| Grade | 1.017173 | 0.745926 | 1.387055 | 0.914313 |
| Stage | 1.86469 | 1.455816 | 2.388397 | 8.07E-07 |
| T | 1.804388 | 1.43414 | 2.270223 | 4.73E-07 |
| M | 3.849834 | 1.206809 | 12.28133 | 0.022752 |
| N | 2.021833 | 0.493927 | 8.276134 | 0.327563 |
| TCP1 | 1.867291 | 1.316752 | 2.648014 | 0.000459 |
| CCT2 | 2.516661 | 1.742488 | 3.634792 | 8.63E-07 |
| CCT3 | 1.927338 | 1.353826 | 2.743803 | 0.000272 |
| CCT4 | 2.598936 | 1.73284 | 3.89792 | 3.87E-06 |
| CCT5 | 2.087706 | 1.515558 | 2.875849 | 6.66E-06 |
| CCT6A | 2.185639 | 1.489106 | 3.207978 | 6.51E-05 |
| CCT6B | 0.317404 | 0.137033 | 0.73519 | 0.007411 |
| CCT7 | 2.460104 | 1.63011 | 3.712701 | 1.81E-05 |
| CCT8 | 2.228138 | 1.468159 | 3.381513 | 0.000167 |

Abbreviations: OS, overall survival time; HR, hazard ratio; CI, confidence interval.

Table S2 Univariate analysis of CCT subunit genes and clinical characteristics in the ICGC cohort.

| **Variables** | **HR** | **95% CI for HR** | | ***P***-value |
| --- | --- | --- | --- | --- |
|  |  | **Lower** | **Upper** |  |
| Gender | 0.518528 | 0.278208 | 0.966438 | 0.038694 |
| Age | 1.001985 | 0.971913 | 1.032987 | 0.89852 |
| Stage | 2.154649 | 1.492864 | 3.109803 | 4.13E-05 |
| Prior Malignancy | 1.750989 | 0.773288 | 3.964839 | 0.179145 |
| TCP1 | 2.673925 | 1.569135 | 4.556571 | 0.000299 |
| CCT2 | 2.05534 | 1.561338 | 2.705642 | 2.80E-07 |
| CCT3 | 2.302287 | 1.403782 | 3.77589 | 0.000954 |
| CCT4 | 4.07178 | 2.302847 | 7.19952 | 1.38E-06 |
| CCT5 | 2.503812 | 1.681686 | 3.72785 | 6.20E-06 |
| CCT6A | 3.917054 | 2.324035 | 6.602016 | 2.96E-07 |
| CCT6B | 0.455575 | 0.226395 | 0.916754 | 0.027554 |
| CCT7 | 4.442866 | 2.570447 | 7.679231 | 9.23E-08 |
| CCT8 | 4.159609 | 2.251369 | 7.685255 | 5.34E-06 |

Abbreviations: OS, overall survival time; HR, hazard ratio; CI, confidence interval.
